# Morphology of the Devonian mandibulate arthropod *Rhyniognatha* and its implications for the timing of insect evolution

**DOI:** 10.1038/s41598-026-70516-3

**Published:** 2026-09-18

**Authors:** Emma J. Long, Gregory D. Edgecombe, Markus Koch, Xiaoya Ma

**Affiliations:** 1 Centre for Ecology & Conservation, University of Exeter, Penryn Campus, Penryn, TR10 9FE Cornwall UK; 2 Science Group, The Natural History Museum, Cromwell Road, London, SW7 5BD UK; 3 Scientific Research, The British Museum, Great Russell Street, London, WC1B 3DG UK; 4 Bonn Institute of Organismic Biology, Section II: Animal Biodiversity, University of Bonn, An der Immenburg 1, 53121 Bonn, Germany

**Keywords:** Rhynie chert, Insect evolution, Mandibulate mouthparts, Hexapoda, Diplura, Symphyla, Evolution, Zoology

## Abstract

Insects are phenomenally diverse, yet their evolutionary origins remain poorly understood. The fossil *Rhyniognatha hirsti* from the Early Devonian Rhynie chert (ca. 408 Ma) has been widely accepted as the earliest insect and a crown-group pterygote (flying insect), based on the interpretation of short, dicondylic mandibles. For over two decades, this fragmentary specimen has served as a key calibration point in insect molecular phylogenies. An alternative hypothesis has proposed a myriapod affinity, citing ocular, antennal and maxillary structures. Here, we re-examine *Rhyniognatha* using confocal laser scanning microscopy. We find no evidence for an anterior mandibular acetabulum, refuting true dicondyly and undermining its placement within Dicondylia (silverfish–pterygote clade). We re-assess the proposed centipede affinity, demonstrating that certain diagnostic features are more plausibly interpreted as exoskeletal folds, and that the mandibles lack a clear myriapod-type base plate. Comparative analysis reveals similarities with apterygote hexapods, including bifurcated maxillary endites and comb-like superlinguae, and myriapods, such as an edafopodan-like elongate suspensorium, but does not support assignment to any specific mandibulate lineage. Consequently, *Rhyniognatha* cannot be placed within crown-group Insecta and is unsuitable as a calibration fossil for insect evolution, shifting the minimum age of winged insects by approximately 80 million years.

**Supplementary Information:**

The online version contains supplementary material available at https://doi.org/10.1038/s41598-026-70516-3.

## Introduction

Few fossils have shaped our understanding of insect evolution as profoundly as *Rhyniognatha hirsti* Tillyard, 1926^1–3^. Known from a single fragmentary specimen in the Lower Devonian Rhynie chert of Scotland, the fossil preserves a pair of mandibles enclosed within a cuticular framework. Since its reinterpretation over two decades ago as the oldest known insect and a crown-group pterygote, *Rhyniognatha* has played a central role in supporting a Devonian–and possibly Silurian–origin of insect flight^2–4^. This interpretation was based on two key features: the presence of dicondylic mandibles, defined by an anterior acetabulum and posterior condyle, and the short, triangular morphology of the mandibles, reminiscent of pterygote insects. However, the fragmentary nature of the specimen has invited continued scrutiny. A competing hypothesis proposed a myriapod affinity, specifically a centipede affinity, reinterpreting supposed apodemes as mandibular glands and describing new features, including putative ocular and antennal sockets, palps, and a pair of ventral comb-like projections interpreted as first maxillae^5^. Although that study did not observe an acetabulum as the anterior mandibular articulation, it also did not definitively exclude its presence, leaving the question of dicondyly in *Rhyniognatha* unresolved. In the absence of a definitive and compelling re-assessment, *Rhyniognatha* has continued to be regarded as a stem-group dicondylian insect and remains widely used as a calibration point in molecular phylogenies, anchoring estimates for the origin of insects and the evolution of flight^6–9^.

**Fig. 1:**
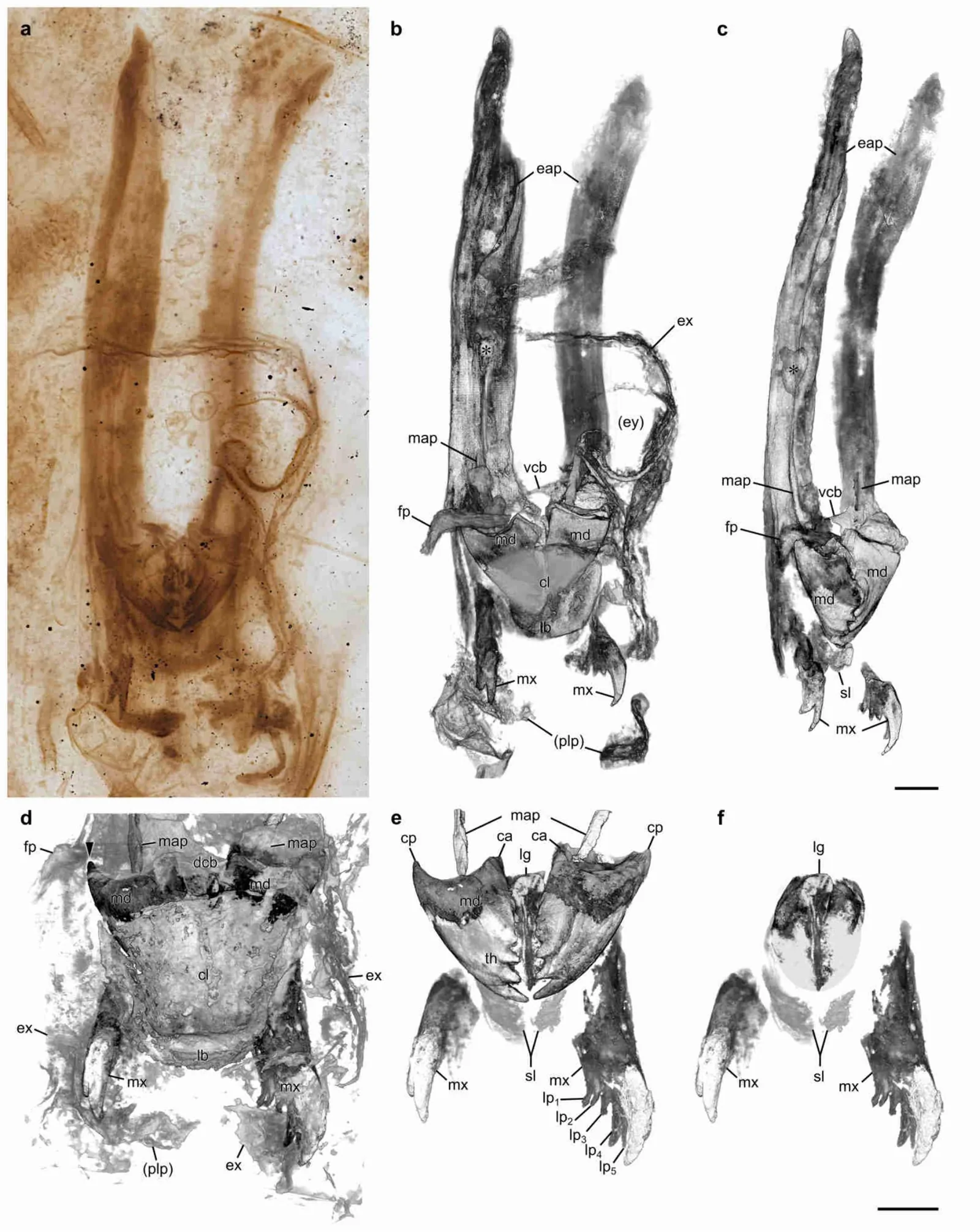
Anatomical overview of *Rhyniognatha hirsti*. Dorsal view, anterior is down (**a**) Light micrograph. (**b–f**) Three-dimensional models reconstructed from confocal laser scanning microscopy (CLSM) data. Elongate paired apodemes are linked ventromedially by cuticular bridges at the base of short, triangular mandibles enclosed dorsally by a clypeo-labrum. Short ridge-like apodemes arise from the mandibles. A black asterisk denotes a cuticular fragment within the left apodemal arm. Bifurcated maxillary endites (laciniae) occur laterally. (**b**) Folded exoskeletal fragments occur dorsally and laterally. Transparent shading indicates the extent of the clypeo-labrum. (**c**,**e**,**f**) Exoskeleton and clypeo-labrum digitally removed. (**c**) Oblique dorsal view depicting superlinguae. (**d–f**) Close-up views of the mouthparts. (**d**) The mandibles exhibit two weak condyles. The posterior condyle of the left mandible (black arrowhead) articulates with a dorsal skeletal element housing the posterior fossa. (**e**) The mandibles bear four teeth. A medial lingua and elongate superlinguae occur ventrally. Laciniae are terminally bifurcated and bear five medial lamella-like processes. (**f**) Mandibles removed to reveal underlying lingua (grey circle indicates approximate extent). The left superlingual arm articulates at the base of the lingua. Abbreviations: ca, anterior condyle; cl, clypeus; cp, posterior condyle; dcb, dorsal cuticular bridge; eap, elongate apodeme; (ey), previously proposed eye socket^5^, not supported here; ex, exoskeleton; fp, skeletal fragment bearing the fossa of the posterior condyle; lb, labrum; lc, lacinia; lg, lingua; lp_1–5_, lamellar process 1–5; map, mandibular apodeme; md, mandible; mx, first maxilla; (plp), previously proposed palp^5^, not supported here; sl, superlingua; th, mandibular teeth; vcb, cuticular bridge. Scale bars, 50 μm.

**Fig. 2:**
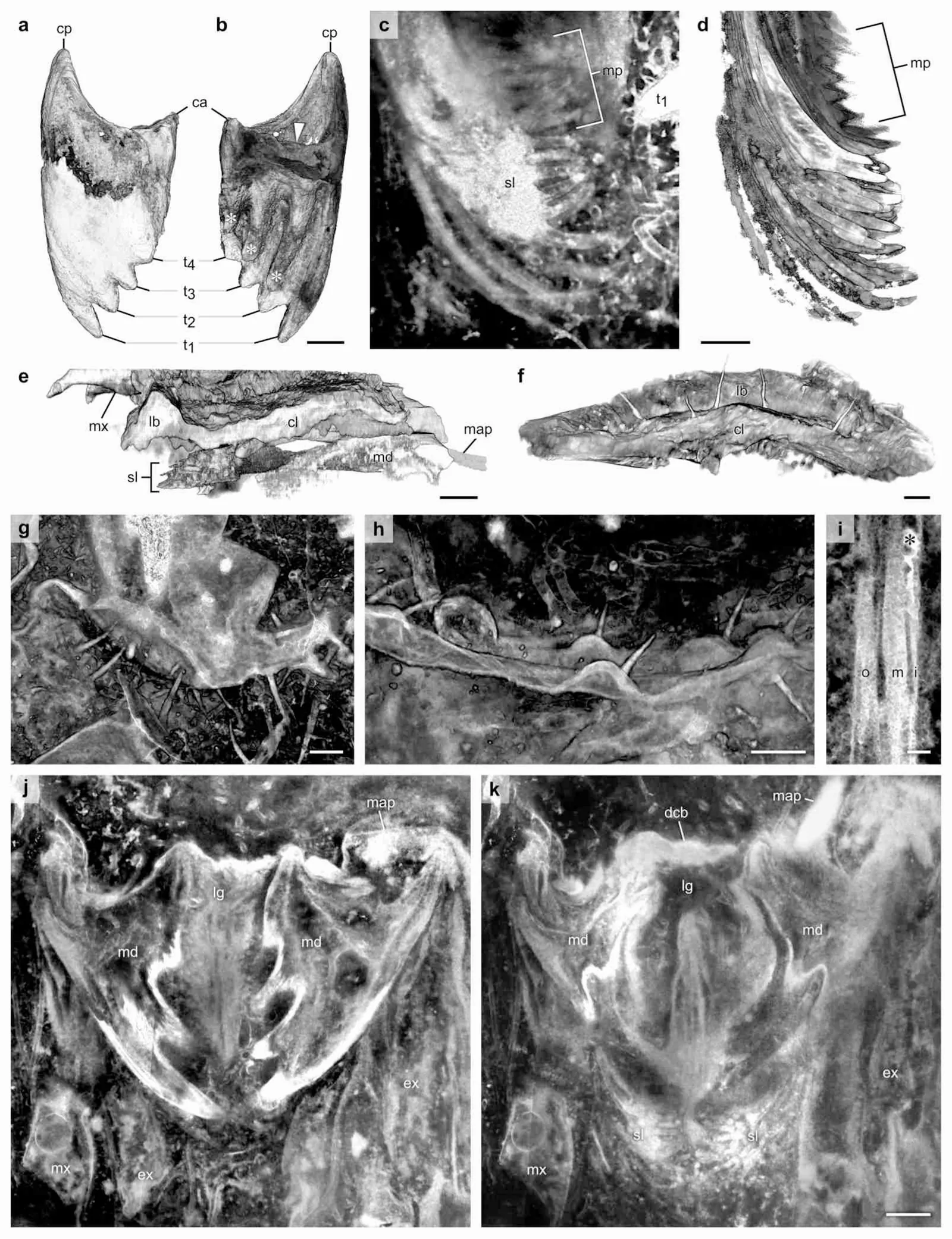
Detailed anatomy of *Rhyniognatha hirsti*. CLSM reconstructions visualised as interpretative models (**a**,**b**,**d–f**) and virtual dissections (**c**,**g–k**). (**a**,**b**) Left mandible showing four teeth and two weak condyles in dorsal (**a**) and ventral (**b**) views. Teeth 2–4 are bicuspate; white asterisks denote ventral cusps. A white arrowhead marks the ventral mandibular base. (**c**,**d**) Left superlingual arm exhibiting a comb-like morphology with elongate lateral processes and shorter medial projections. (**e**) Lateral view in section. The clypeo-labrum occurs dorsally. The truncated upper margin reflects the plane of thin sectioning. (**f**) Clypeo-labrum in posterior perspective. A row of clypeal setae marks the boundary between clypeus and labrum. (**g**,**h**) Exoskeletal folds exhibiting tuberculate and spine-like surface sculpture. (**i**) Detail of the left apodemal arm showing outer, medial and inner endoskeletal layers. A black asterisk indicates a cuticular element between the medial and inner layers. (**j**,**k**) Sectioned views of the hypopharynx, mandibles and maxillae. The lingua bears a medial lip, and the comb-like superlinguae extend beyond the tips of the overlying mandibles. The right mandibular apodeme is shown arising from the mandibular base. Abbreviations: ca, anterior condyle; cl, clypeus; cp, posterior condyle; dcb, dorsal cuticular bridge; ex, exoskeleton; i, inner elongate apodeme layer; lb, labrum; lg, lingua; m, medial elongate apodeme layer; map, mandibular apodeme; md, mandible; mp, medial projections of the superlingua; mx, (first) maxilla; o, outer elongate apodeme layer; sl, superlingua; t1, first mandibular tooth. Scale bars: 20 μm (**a**,**b**,**e**,**j–k**), 10 μm (**c**,**d**,**f–i**).

**Fig. 3:**
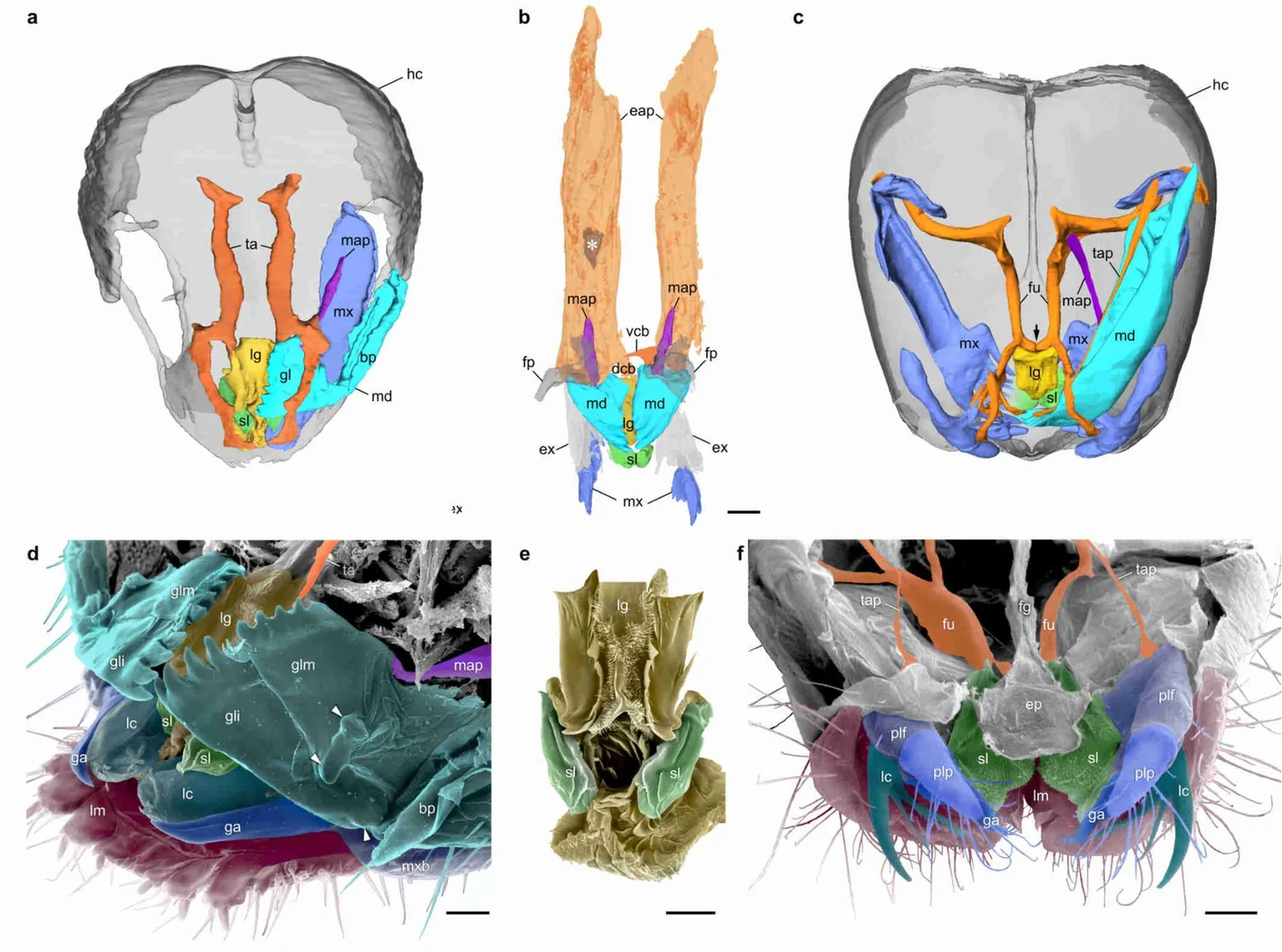
Comparative head anatomy of Symphyla, *Rhyniognatha hirsti* and Diplura. (**a–c**) Three-dimensional reconstructions. Suspensorial sclerites coloured orange for functional comparison, homology not implied. (**a**) *Scutigerella immaculata* (Symphyla), serial-section reconstruction showing tentorial arms, mandible with gnathal lobe and base plate, mandibular apodeme, first maxilla and hypopharynx with lingua and superlinguae. (**b**) *Rhyniognatha hirsti*, CLSM. Bridged elongate apodemes, mandibles, mandibular apodemes, maxillary endites (laciniae) and hypopharynx with lingua and superlinguae. An asterisk denotes a miscellaneous cuticular element. (**c**) *Heterojapyx* sp. (previously identified as *Atlasjapyx*^21^) (Diplura), synchrotron-µCT. Fulturae linked by cuticular bridge (arrow), mandible, mandibular apodeme, anterior tentorial apodeme (originating from suspensorial sclerites), first maxillae and hypopharynx with lingua and superlinguae (left arm digitally faded). (**d–f**) Scanning electron micrographs. (**d**,**e**) *Scutigerella causeyae* (Symphyla). (**d**) Mandibular gnathal lobe with incisor and molar regions. Paired arrowheads indicate condylic ridge. Single arrowhead marks articulation with base plate. (**e**) Hypopharynx with lingua and superlinguae. (**f**) *Catajapyx aquilonaris* (Diplura), mandibles removed. Lingua (not visible) lies below the epipharynx. Abbreviations: bp, mandibular base plate; cl, clypeus; dcb, dorsal cuticular bridge; eap, elongate apodeme; ep, epipharynx; ex, exoskeleton; fg, foregut; fu, fulturae; ga, galea; gl, mandibular gnathal lobe; gli, incisor region of gnathal lobe; glm, molar region of gnathal lobe; lb, labrum; lc, lacinia; lg, lingua; lm, labium; map, mandibular apodeme; md, mandible; mx, first maxilla; mxb, base of mx; plf, palpifer of mx; plp, palp of mx; sl, superlingua; ta, tentorial arm; tap, anterior tentorial apodeme; vcb, ventral cuticular bridge. Scale bars: 50 μm (**b**,**f**); 20 μm (**d**,**e**).

Here, we re-examine the holotype specimen of *Rhyniognatha hirsti* (NHMUK PI In 38234) using confocal laser scanning microscopy and digital reconstruction (Figs. 1b–f, 2, 3b and S1c). These techniques exploit the fossil’s exceptional three-dimensional preservation and enable a more detailed assessment than was previously possible using transmitted light microscopy^1–3,5,10^ (Figs. 1a and S1a,b).

**Fig. 4:**
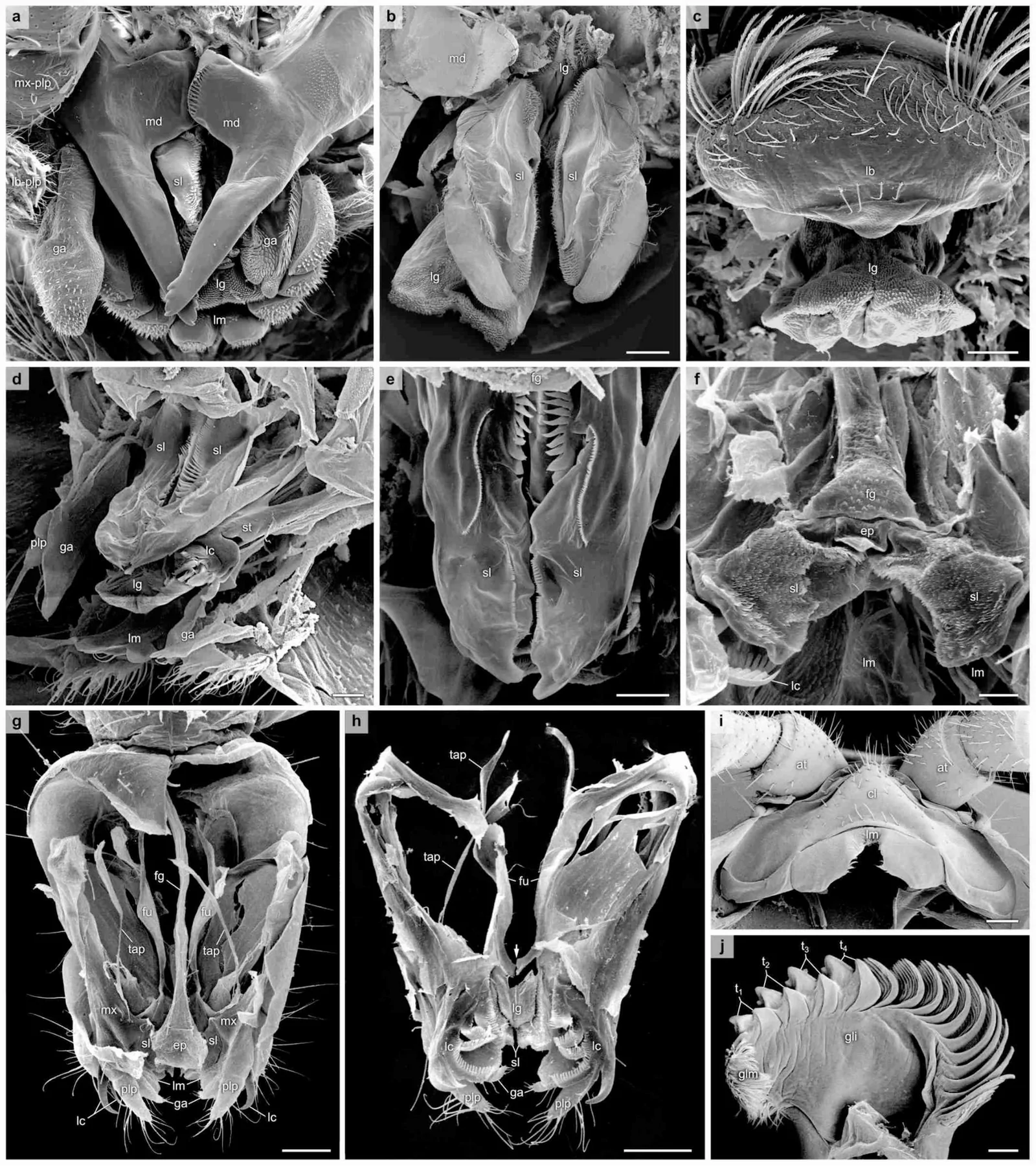
Head structures of extant apterygote hexapods and myriapods. Scanning electron micrographs. (**a**,**b**) *Petrobius brevistylis* (Archaeognatha). (**a**) Overview showing monocondylic mandibles, first maxillae and hypopharynx with lingua and elongate superlinguae. (**b**) Hypopharynx. (**c**) *Ctenolepisma longicaudata* (Zygentoma). Setose labrum and hypopharynx comprising a lingua (no superlinguae). (**d**,**e**) *Tomocerus minor* (Collembola). (**d**) Overview depicting maxillae and hypopharynx with lingua and elongate superlinguae. (**e**) Superlinguae. (**f–h**) *Catajapyx aquilonaris* (Diplura). (**f**) Hypopharynx with lingua (obscured by foregut and internal epipharyngeal wall) and superlinguae. (**g**) Overview showing fulturae, first maxillae with palp, galea and lacinia, anterior tentorial apodemes and elongate superlinguae in dorsal view. Mandibles removed. (**h**) Ventral view showing fultural arms linked by a cuticular bridge (white arrow) at the base of the hypopharynx and the lamellar processes of the laciniae. **i**,**j**) Chilopoda. **i**) *Henicops milledgei*. Clypeal and labral regions demarcated by a suture and a row of clypeal setae. (**j**) *Cryptops spinipes*. Mandibular gnathal lobe with reduced molar region and enlarged incisor region bearing four bicuspate teeth. at, antenna; cb, cuticular bridge; cl, clypeus; fg, foregut; fu, fulturae; ga, galea; gli, incisor region of mandibular gnathal lobe; glm, molar region of mandibular gnathal lobe; lb, labrum; lc, lacinia; lg, lingua; lm, labium (second maxilla); md, mandible; mx, first maxilla; plf, palpifer of mx; plp, palp (mx where unspecified); sl, superlingua; st, stipes; t_1–4_, mandibular teeth 1–4; tap, anterior tentorial apodeme. Scale bars: 100 μm (**a–b**,**g**,**h**); 20 μm (**c–f**); 200 μm (**i**); 50 μm (**j**).

Using this approach, we re-assess the morphology and articulation of the mandibles and associated head structures, comparing *Rhyniognatha* with Symphyla and Diplura, representative myriapod and primitively flightless hexapod taxa, respectively. These groups are selected because they retain relatively conservative head architectures that bracket key variation in mandibular articulation, endoskeletal organisation, and hypopharyngeal morphology^11^. Symphylans possess elongate mandibular apodemes and a complete hypopharynx with reduced superlinguae (Fig. 3a,d,e), whereas diplurans exhibit differentiated maxillary endites and superlinguae (Figs. 3c, f and 4f–h) but lack the derived mandibular articulations and head specialisations of ectognathous hexapods^11^. Together, these taxa provide a comparative framework for testing competing hypotheses of affinity that have constrained our understanding of the origin of insects and the early evolution of flight in the fossil record.

## Results

### Mandibles

The preserved mouthpart complex is interpreted as orthognathous, consistent with the directional terminology used in previous descriptions of *Rhyniognatha*^2,3,5^ and provides the framework for the anatomical description below. However, the fragmentary preservation of the fossil, and the absence of a complete head capsule, preclude a definitive assessment of overall head posture. Our 3D reconstruction of *Rhyniognatha* reveals symmetrical, short, triangular mandibles, each bearing four teeth (contra two^1^, three^5^ and five^2,3,10^) that carve ridges into the medial mandibular surface (Figs. 1a,c,e, 2a,b,j, 3b and S1d). The distal tooth (t1) is unicuspate, whereas the remaining three teeth (t2–4) are bicuspate (Fig. 2a,b). Both anterior and posterior condyles articulate with dorsal skeletal elements (Figs. 1b–e and 3b; see Skeletal framework and cuticular structures), but these condyles are relatively weak and lack the robust, ball-and-socket articulation typical of pterygote dicondyly ^2,3,11,12^. Although the short, triangular morphology of the mandibles is reminiscent of metapterygote insects^2,3^, we find no evidence for an anterior acetabulum or a molar grinding surface on the inner mandibular edge (Figs. 1e and 2a,b).

### Clypeo-labrum

Dorsal to the mandibles lies a thin, relatively well-defined plate-like structure with a distinct rim approximately twice the thickness of the main plate (Figs. 1b,d, 2e,f and S1d). A marginal row of curved setae delimits the boundary between the plate and the thickened rim (Fig. 2f). Based on its morphology and position relative to the mandibles^11^, we interpret this structure as the clypeo-labrum. The posterior margin may represent a fracture along a structural boundary rather than a true frontoclypeal sulcus or epistomal ridge, a feature considered apomorphic for Dicondylia^11^ (Fig. 1d).

### Maxillae

Ventral to the mandibles lies a pair of appendages with terminally bifurcated endites, the inner prong slightly longer than the outer (Figs. 1, 2e, 3b and S1d). This morphology, previously described only unilaterally^2,3^, and hypothesised to be paired^13^, is herein confirmed as bilateral. We identify these appendages as maxillae, consistent with earlier interpretations^2,3^. Unlike previous reconstructions, which conflated these structures with adjacent cuticular folds interpreted as laciniae^2,3^ or palps^5^, the distally bifurcated maxillary endites are clearly discrete (Fig. 1b,d). A medial branch occurs basal to the terminal bifurcations, the medial face of which bears a single row of five slender, lightly sclerotised, lamella-like projections on the right maxilla (Figs. 1b–f and S1d). We interpret this branch as the lacinia, and its projections as lamellar processes. A corresponding palpifer–galea–palp complex (coxal endite 2) is not preserved. Each maxilla can be traced posteriorly to at least the level of the mandibles, where they appear to bend out of the plane of the section (Figs. 1b–f and 2j, k). No regular lacinial setae are apparent, although this may reflect the preservational limits or scan resolution.

### Hypopharynx

Immediately ventral and medial to the mandibles lies a lightly sclerotised, roughly ovate plate from which two anterolateral, comb-like arms project just beyond the tips of the mandibles (Figs. 1c,e,f, 2c–e,k, 3b and S1d). We interpret this structure as the hypopharynx, comprising a central lingua and paired superlinguae (contra interpretation as first maxillae^5^). The lingua exhibits medial sclerotisation, forming a pair of “lips” (Figs. 2j,k and S1d). The superlinguae arise posterolaterally and curve anteromedially (Figs. 1f, 2c,d,k and S1d), each forming a comb-like structure bearing elongate lateral projections and shorter, tapered medial elements (Fig. 2c,d).

### Skeletal framework and cuticular structures

The mandibles, maxillae, hypopharynx and clypeo-labrum are embedded within a skeletal framework of variably sclerotised, deformed and folded elements (Figs. 1, 2e,g–k, 3b and S1d; see Preservation). Skeletal regions can be categorised as exoskeletal or endoskeletal based on a combination of surface sculpture, structural context and spatial relationships. Exoskeletal cuticle commonly exhibits setose/spinose and tuberculate ornamentation (Fig. 2g,h). Skeletal elements associated with the posterior articulation of the mandibles, including those housing the fossae of the anterior and posterior condyles, lack conspicuous ornamentation but are interpreted herein as exoskeletal based on their position and dorsal placement relative to the mandibles (Figs. 1b–d, 3b and S1d). Elongate skeletal arms (Figs. 1a–c, 2i, 3b and S1d), previously interpreted as mandibular glands^5^ or components of the head capsule^3^–possibly apodemes^2^–are reinterpreted here as multilayered endoskeletal structures, or apodemes, the latter used here in a broad sense to denote internal cuticular projections, including ridge-like phragmata, without presupposing invagination. The apodemal arms lack external ornamentation and form a multilayered framework comprising at least three discrete layers (Fig. 2i). The innermost layer forms dorsal and ventral cuticular bridges at the base of the hypopharynx and mandibles (Figs. 1b–d, 2k, 3b and S1d) and is separated from the outer layer by a densely sclerotised, ridge-like structure arising from the mandibular base, possibly corresponding to a mandibular apodeme (Figs. 1b–e, 2e,j,k, 3b and S1d).

The apodemal arms exhibit localised variation in apparent density, particularly along their lateral margins (Fig. 1a–c). This pattern does not correspond to discrete anatomical boundaries and likely reflects local increases in cuticle thickness resulting from overlap, folding, or compression.

An additional sclerotised structure is present within the left apodemal arm, although its homology remains uncertain (Figs. 1b,c, 2i, 3b and S1d). Prior interpretations of ocular and antennal sockets^5^ are reinterpreted here as asymmetrical exoskeletal folds that extend anterolaterally beyond the level of the maxillary endites (Fig. S1a,b) and posteriorly beyond the previously proposed head capsule boundary (Fig. 1b).

### Preservation

*Rhyniognatha* is not uniformly preserved; the autofluorescence signal, considered a proxy for material density or the degree of sclerotisation^14,15^, varies significantly between structures (e.g. see the disparate autofluorescence signatures of the mandibles and lingua in Fig. 2j,k). The most highly autofluorescent structures, corresponding to the most densely sclerotised regions, appear dark brown to black under brightfield illumination (Figs. 1a and S1a,b). These include the distal tips of the mandibular teeth (e.g. Figs. 1a and 2j,k), the maxillary endites (e.g. Fig. 1a,e,f), and the ridge-like apodeme arising from the mandibular base (e.g. Fig. 2j,k). *Rhyniognatha* is preserved in a heterogeneous, clotted chert matrix with a mulm-like texture consistent with aqueous silicification^16^, in association with fragmentary arthropod remains (Figs. 1a and S1).

## Discussion

The singular nature and disjointed preservation of *Rhyniognatha hirsti* have long complicated its phylogenetic placement. However, its Early Devonian age makes an accurate interpretation crucial for reconstructing and dating the early evolution of insects.

The latest contribution in a century-long history of study proposed a myriapod affinity based on a re-interpretation of the fossil’s mandibular morphology and the identification of maxillary, glandular, ocular, and antennal structures^5^. Our re-examination of *Rhyniognatha* using confocal imaging does not support these interpretations. Putative ocular and antennal sockets, used to suggest a scutigeromorph centipede affinity^5^, are instead asymmetrical folds of ornamented exoskeleton rather than sensory structures (Fig. 1a,b,d). Paired elongate structures associated with the mandibles and interpreted as centipede-like ectodermal glands^5^ share the preservational and optical properties of surrounding cuticular elements (Fig. 1a–c). These structures lack evidence of a central lumen, terminal branching, or associated glandular tissue, and their surface morphology does not resemble a glandular reservoir^17^. Instead, their texture and structural continuity are consistent with internal cuticular reinforcements, supporting their interpretation as apodemes^2^ rather than glandular ducts. Although homologues among extant taxa remain uncertain, these elongate apodemes are comparable to the suspensorial sclerite frameworks of myriapods^11,18–20^ (tentorial arms; Fig. 3a) and entognathous hexapods^11,21,22^ (fulturae; Figs. 3c and 4g,h). We herein use the term “suspensorium” in a broad functional sense for the cuticular framework supporting the mandibular and hypopharyngeal complex, and “fultura-like” for structures resembling sclerotic extensions of the suspensorium called fulturae in entognathan hexapods and Archaeognatha, without implying direct homology (see Methods: Terminology). The elongate apodemes of *Rhyniognatha* are unusually long and have no exact equivalent among the comparative taxa examined herein. This may represent a distinctive condition of *Rhyniognatha*, although the apparent posterior extent of these structures should be interpreted with caution due to the fragmentary nature of the material and uncertainty regarding the posterior boundary of the head.

In *Rhyniognatha*, a short, ridge-like mandibular apodeme lies medial to, and is partially enclosed by, the paired elongate apodemes (Figs. 1b–e,3b and S1d). This spatial relationship is reminiscent of the gliding channel in Symphyla, in which the mandibular apodeme runs in close apposition to, and interacts closely with, the anterior tentorial arms within a mechanically integrated suspensorial framework^23,24^ (Fig. 3a,d). In *Rhyniognatha*, the mandibular apodeme extends between the medial and inner layers of the elongate apodemal arms, forming a nested configuration that suggests a comparable structural relationship. This arrangement reinforces the interpretation of the elongate apodemes as integrated components of an endoskeletal support system.

The structural integration of these elements is further reinforced by cuticular bridges linking the bases of the paired elongate apodemes (Figs. 1b–d, 2k, 3b and S1d). In most myriapods, the medial connection between the tentorial arms is ligamentous rather than cuticular, comprising one or several collagenous tendons that coalesce to varying degrees into a transverse ligament^20,24^. By contrast, a cuticular anterior bridge links the tentorial arms in Pauropoda^25^. In entognathous hexapods, the suspensorial framework is likewise linked by a cuticular bridge formed by the fultural arms at the base of the hypopharynx (Figs. 3c and 4g,h). The presence of a cuticular interapodemal bridge in *Rhyniognatha* suggests closer correspondence with the cuticularised hexapod and pauropod conditions than with the ligamentous architecture typical of other myriapods. This overall configuration most closely resembles the symphylan condition, with medially bridged suspensorial arms accommodating the mandibular apodeme in a gliding channel. Elongation and anterior cuticular bridging of the tentorial arms, features also observed in the elongate suspensorial apodemes of *Rhyniognatha*, are also found in Pauropoda^25^. The endoskeletal anatomy of *Rhyniognatha* thus recalls character combinations discussed in the context of Edafopoda (Symphyla + Pauropoda).

Hexapod affinities are suggested by the organisation of the hypopharynx. Paired, elongate, comb-like structures previously interpreted as chilopod-like first maxillae^5^ are here identified as hexapod-like superlinguae, associated with a faintly preserved medial body, the lingua (Figs. 1c,e,f, 2c–e,k, 3b and S1d). Elongate superlinguae comparable to those in the fossil are present in Archaeognatha^26,27^ (Fig. 4a,b), Collembola^28,29^ (Fig. 4d,e) and Diplura (Figs. 3c,d and 4f–h), although these groups lack comb-like projections. A complete hypopharynx with paired superlinguae is also observed in Symphyla^26,30^ and Pauropoda^31^, although the superlinguae are markedly reduced (Fig. 3a,d,e). In Chilopoda, only a simple lingua is present^11,26^. Superlinguae are absent in Zygentoma (Fig. 4c) and in most Pterygota, in which the hypopharynx primarily consists of a tongue-like lingua projecting into the pre-oral chamber^32,33^ although further reduction or modification may occur depending on feeding mode. Among pterygotes, paired, elongate, comb-like superlinguae comparable to those of *Rhyniognatha* are observed only in some larval Ephemeroptera^11,34^.

The clypeus of *Rhyniognatha* forms a well-defined, laterally discrete external sclerite (Figs. 1b,d, 2e,f and S1d) most closely resembling the condition observed in early-branching ectognathous hexapods such as Archaeognatha^11^ and Zygentoma^29,35^. A marginal row of clypeal setae occurs along the clypeo-labral margin (Fig. 2f), a feature also observed in some myriapods (Fig. 4i). By contrast, the clypeus typically lacks distinct sutures or marginal rims in most myriapods^11,36^ and entognathous hexapods^11^, such that its lateral boundaries are poorly delimited. Where present in these groups, demarcation is restricted to the clypeo-labral boundary. Among entognathous hexapods, Diplura (Figs. 3f and 4g) exhibit a labrum of comparable relative size to that of *Rhyniognatha* (Figs. 1b,d and 2e,f), but the clypeus remains laterally indistinct.

The endites of the first maxillae in *Rhyniognatha* are terminally bifurcated (Figs. 1, 2e, 3b and S1d), a condition not observed in Myriapoda^11^ (Fig. 3d). In Collembola and Archaeognatha, the first maxillary endites terminate in globular or claw-like processes rather than forming distinct apical branches^11,28^ (Fig. 4a). In Diplura (Figs. 3c,g and 4g,h), the lacinia bears mesally directed lamellar processes but does not form a distally bifurcated, tapering structure^11,37^. These lamellae are hyaline and flexible, varying in shape from straight to comb-like. Their arrangement on the lacinia ranges from a single row, as in the comb-like lamellae of japygoid diplurans^11^ (Fig. 4h), to closely appressed, compact (fist-like) configurations in collembolans. The maxillary morphology of *Rhyniognatha* is therefore more consistent with patterns of endite differentiation in some early-branching hexapods than with those of myriapods.

The mandible of *Rhyniognatha* is notable for comprising only a gnathal lobe, with no clear evidence of a base plate (Figs. 1a–e, 2a,b, 3b and S1d). In myriapods, the base plate occurs either as a discrete sclerite to which the gnathal lobe articulates, as in Diplopoda^38^ and Symphyla^20^ (Fig. 3a), or as a structure continuous with the independently musculated gnathal lobe, as in Chilopoda^39^. This mandibular division represents one of the strongest autapomorphies of Myriapoda. The structure associated with the mandibular articulation, here interpreted as housing fossae for the mandibular condyles, could alternatively be considered a remnant of a base plate (Figs. 1b–d, 3b and S1d). However, this interpretation would require selective loss or fragmentation of a structurally integrated mandibular element, while the adjacent gnathal lobe and associated structures remain *in situ* and well preserved. Although taphonomic loss of a base plate cannot be excluded, given the incomplete preservation of parts of the head capsule and other mouthparts (such as the cardo, stipes, and palp), invoking such loss specifically to account for the absence of a base plate would be *ad hoc*.

In *Rhyniognatha*, each mandible bears three bicuspate teeth and a distal unicuspate tooth, with the cusps arranged in two overlying rows along the mandibular axis (Fig. 2a,b). The configuration of teeth closely matches that of the gnathal lobe in Chilopoda (Fig. 4j). Both anterior and posterior mandibular condyles are present, yet the anterior articulation is weak and lacks a true ball-and-socket joint (Figs. 1b–e, 2a,b, 3b and S1d). A similar arrangement of dual articulations is also observed in myriapods, in which the posterior condyle articulates with the mandibular base plate and the anterior condyle with elements of the tentorium^11,39^. In *Rhyniognatha*, the posterior condyle appears to articulate with dorsal exoskeletal elements, which may represent the head capsule (Figs. 1b–d, 3b and S1d), although the identity of these structures cannot be determined unambiguously. The resulting combination of features overlaps with both myriapod and hexapod conditions, and the presence of dual articulations alone is not diagnostic. However, the absence of a well-defined anterior acetabulum, together with the overall weakness of the articulation, argues against obligate dicondyly. A weakly developed dual articulation is consistent with the facultative dicondyly observed in Zygentoma^11,22^ and larval Ephemeroptera^40,41^, rather than the obligate dicondyly characteristic of Odonata and Neoptera^11,37^.

The homology and evolutionary polarity of mandibular articulations in Hexapoda remain debated^42–44^. Dicondyly has recently been proposed as a plesiomorphy of Hexapoda based on comparisons with malacostracan crustaceans^43^. However, broader comparative evidence, including the monocondylic mandibles of Diplura, supports monocondyly as the ancestral hexapod condition, with dicondyly arising independently in multiple lineages^44^. Despite this potential convergence, obligate dicondyly remains a useful diagnostic feature for crown-group Dicondylia. Within this framework, the mandibular articulation of *Rhyniognatha*, which lacks an anterior acetabulum, is inconsistent with placement within Dicondylia.

Additional head characters further restrict its phylogenetic position. *Rhyniognatha* retains well-developed superlinguae, a condition inconsistent with crown-group Dicondylia, in which superlinguae are lost or strongly reduced in Zygentoma and most Pterygota. The apparent absence of anterior tentorial apodemes excludes placement within the crown-group Hexapoda. The lack of a corpotentorium, formed by fused anterior and posterior tentorial apodemes forming an X-shaped endoskeleton, argues against placement within Pterygota, while the presence of paired fultura-like elements interconnecting the hypopharyngeal suspensorium with the posterior tentorial apodemes argues against Dicondylia. Fusion of the posterior tentorial apodemes into an unpaired plate also occurs in Archaeognatha, but these insects retain paired fulturae connecting this plate to the hypopharyngeal suspensorium. By contrast, the fulturae are reduced in Dicondylia.

The combination of weakly developed dual mandibular articulations, conservative endoskeletal architecture, well-developed comb-like superlinguae and bifurcated maxillary endites places *Rhyniognatha* outside crown-group Dicondylia, but does not unambiguously resolve its affinity between early hexapods and myriapods. Chronograms that previously used *Rhyniognatha* to constrain the minimum age of Dicondylia (and Insecta) therefore significantly overestimate divergence times^45^. Removing *Rhyniognatha* as a crown-Dicondylia calibration fossil fundamentally revises the inferred timing and sequence of key hexapod innovations, notably obligate dicondylic mandibles and, by implication, wings, and implies an approximately 80-million-year shift in the minimum ages inferred for insect origins and insect flight.

## Methods

### Terminology

We use the term “suspensorium” in a broad descriptive sense for exoskeletal and endoskeletal elements that support the mandibular and hypopharyngeal complex. In extant entognathous hexapods and in Archaeognatha, this hypopharyngeal suspensorial system comprises sclerotised extensions called fulturae, or lingual stalks, which partially extend into ridge-like phragmata and thereby provide sites for the attachment of extrinsic muscles of the first maxillae. In Archaeognatha, these phragmata contribute, together with the posterior tentorial apodemes, to the formation of a large, unpaired, plate-like posterior tentorium. In restricting the term “fulturae” to these extensions of the hypopharyngeal suspensorium, we follow the terminology used by the classic French entomologists Jacques Bitsch, Jean François, and Jean Chaudonneret in detailed studies of the head morphology of apterygote hexapods. Because homology between the elongate apodemes of *Rhyniognatha* and the fulturae of extant hexapods cannot be demonstrated, we use “fultura-like” only to describe structures that resemble fulturae in position and configuration.

### Brightfield microscopy

*Rhyniognatha hirsti* was imaged under brightfield illumination at the Digitisation and Microscopy Laboratory (Imaging and Analysis Centre), Natural History Museum, London, using an Olympus DP73 camera (Olympus, Tokyo, Japan) attached to an Olympus BX63 microscope. Images were captured using Olympus cellSens Dimension software version 1.15 (https://evidentscientific.com/en/products/software/cellsens) as z-stacks with 2× (Fig. S1a), 20× (Fig. S1b), and 40× (Fig. 1a) objectives. Each z-stack was processed into a single extended depth of focus image using rendering method C in Helicon Focus Pro software version 6.3.0 (https://www.heliconsoft.com/heliconsoft-products/helicon-focus/).

### Confocal laser scanning microscopy

Confocal image stacks of *Rhyniognatha hirsti* were acquired at the Imaging and Analysis Centre, Natural History Museum, London, using a Nikon A1-Si Confocal Laser Scanning Microscope (CLSM) attached to a Nikon Eclipse E upright microscope. Autofluorescence was excited with a 640 nm laser (Coherent Inc., USA), and emitted fluorescence was detected using a photomultiplier tube within a 675–725 nm emission window. Signal-to-noise ratio was improved using line averaging (8× or 16×) and the pinhole was set to 30.7 μm. Imaging was performed with a 40× Plan Fluor oil-immersion objective lens. For the overview scan of the fossil, multiple overlapping z-stack tiles were acquired and automatically stitched in Nikon NIS Elements, version 4.60 (https://www.microscope.healthcare.nikon.com/products/software/nis-elements). Detailed scan parameters are provided in Table S1 and in the individual MorphoSource records^46^.

### Scanning electron microscopy

Specimens of *Henicops milledgei* Hollington & Edgecombe, 2004 (MV NOH-1779) (Fig. 4i) and *Cryptops spinipes* Pocock, 1891 (Fig. 4j) were dissected, air-dried, sputter-coated with gold using an Emitech K550 ion coater and imaged at 20 kV on a ZEISS LEO 435VP scanning electron microscope equipped with a Robinson backscatter detector at the Australian Museum. The SEM stubs are deposited in Museums Victoria (MV), Melbourne, and the Australian Museum, Sydney. Specimens of *Catajapyx aquilonaris* Silvestri, 1929 were dissected and macerated in 1–2% pepsin and treated with 37% HCl. In one preparation (Figs. 3f and 4f,g), the dorsal cranial wall (including the clypeo-labrum) and both mandibles were removed; in a second (Fig. 4h), the first maxillae–hypopharynx–fulturae complex was isolated. Specimens of *Tomocerus minor* Lubbock, 1862 were fixed in Bouin’s solution. The head capsule (including the clypeo-labrum) and mandibles were removed to expose the hypopharynx and laciniae of the first maxillae (Fig. 4d,e). Samples of *Catajapyx* and *Tomocerus* were critical-point dried, sputter-coated with gold, and imaged at ~ 20 kV using a CAMSCAN DV4 scanning electron microscope. Specimens of *Scutigerella causeyae* Michelbacher, 1942 (Fig. 3d,e), *Petrobius brevistylis* Carpenter, 1913 (Fig. 4a,b), and *Ctenolepisma longicaudata* Escherich, 1905 (Fig. 4c) were fixed in Bouin’s solution, dissected, critical-point dried using a Balzers CPD 030, and sputter-coated with gold using a Balzers Union SCD 040. Specimens were examined using a FEI Quanta 200 scanning electron microscope at 15–20 kV.

### 3D visualisation and analysis

Three-dimensional data were analysed in Avizo 3D 2025.1 (Thermo Fisher Scientific, USA; https://www.thermofisher.com/uk/en/home/electron-microscopy/products/software-em-3d-vis/avizo-software.html). CLSM image stacks were visualised using ‘standard’ volume rendering of the greyscale data under diffuse lighting. ‘Edge 2D’ and ‘Edge 3D’ functions were applied to enhance fine details. Virtual dissections were performed using the ‘slice’ tool in optimally oriented planes. In some instances, two slices were applied to a single model (Fig. 2j,k). Structures were semi-manually segmented (Segmentation Workroom) using a combination of the ‘magic wand’ tool (to select defined greyscale ranges corresponding to autofluorescent signal intensity) and the ‘paint brush’ tool for manual refinement and subjective delimitation of adjacent or continuous elements. For volumetric visualisation of segmented elements, a ‘mask’ was applied to the segmented region, allowing internal greyscale (autofluorescence) variation to be visualised. Colour maps and opacity levels were adjusted individually for each structure. For the coloured interpretative model (Fig. 3b), segmented structures were rendered as surfaces using unconstrained smoothing. Final renders were assembled as layers in the GNU Image Manipulation Program (GIMP), version 3.0.6 https://www.gimp.org/downloads/). In selected figures, additional segmentation was performed in GIMP to clarify the extent of specific structures, including the clypeus (Fig. 1b), the lingua (Fig. 1f), and the anterior tentorial arm and mandibular apodeme (Fig. 3c). The 3D reconstruction of the symphylan head is adopted from Szucsich et al.^20^ with modified colours for cuticular structures. The dipluran head reconstruction is adopted from Blanke & Machida^21^ with modified colours and corrected interpretations of the maxillary endites and separately visualised apodemes subsumed under muscles by these authors.

## Supplementary Information

Below is the link to the electronic supplementary material.

Supplementary Material 1

## Data Availability

The holotype of *Rhyniognatha hirsti* (NHMUK PI In 38234) is housed at the Natural History Museum, London, UK. Confocal laser scanning microscopy (CLSM) data generated for *R. hirsti* are available on available on MorphoSource: https://www.morphosource.org/projects/000699249?locale=en. Previously published three-dimensional datasets used for comparative visualisation are available in the supplementary materials or repositories associated with their original publications.
