# Supplementary material for: Morphology of the Devonian mandibulate arthropod Rhyniognatha and its implications for the timing of insect evolution
Source: Sci Rep. 2026 Sep 18;16:37059. doi: 10.1038/s41598-026-70516-3 (PMC13739751; doi:10.1038/s41598-026-70516-3)
Supplement: Supplementary file 1 [file 41598_2026_70516_MOESM1_ESM.docx]

Supplementary Information

**Morphology of the Devonian Mandibulate Arthropod *Rhyniognatha* and Its Implications for the Timing of Insect Evolution**

Emma J. Long^1,2,3^, Gregory D. Edgecombe^2^, Markus Koch^4^ & Xiaoya Ma^1^

**Fig. S1 | Contextual overview of *Rhyniognatha hirsti.* a**,**b**, Light micrographs showing the mulm-like texture of the surrounding chert matrix and associated fungal and arthropod fragments. Inset in (**a**) indicates the region enlarged in (**b**). **b**, Black arrowhead marks extreme anterolateral unravelling of the exoskeleton. **c**, Rotated three-dimensional model showing *Rhyniognatha in situ* with nearby arthropod fragments. **i**,**ii**, Close-ups of adjacent arthropod fragments bearing four setae and/or setal sockets, each flanked by two spines (white circles). **d**, Diagrammatic illustration of *Rhyniognatha* in dorsal view. Asterisk denotes miscellaneous cuticular element. Abbreviations: af, arthropod fragment; cl, clypeus; dcb, dorsal cuticular bridge; eap, elongate apodeme; ff, fungal fragment; lb, labrum; lg, lingua; map, mandibular apodeme; md, mandible; mx, first maxilla; se, seta; sl, superlingua; so, setal socket; vcb, ventral cuticular bridge. Scale bars: 500 µm (**a**,**c,d**); 200 µm (**b**), 10 µm (**ci**,**cii**).

**Table S1 | Confocal laser scanning microscopy (CLSM) acquisition parameters for imaging *Rhyniognatha hirsti*.** All scans were conducted using a Plan Fluor 40x Oil DIC H N2 objective, with excitation at 640.4 nm, a z-step size of 0.25 μm, and a confocal pinhole aperture of 30.7 μm.

| *Figure(s)* | *Digital zoom* | *XY pixel dimensions (pixels/µm)* | *No. Z steps* | *XY tile scan size (pixels)* | *Scan depth (µm)* | *Line avg.* | *Pixel dwell time (µs)* | | *XY scan area (tiles)* | *XY tile overlap (%)* | *Morphosource doi* |
| --- | --- | --- | --- | --- | --- | --- | --- | --- | --- | --- | --- |
| Figs. 1b,c, 2 g–i, 3b & S1c | X 1 | 0.31 | 549 | 1024  x 1024 | 137.00 | 8X | | 6.1 | 4 x 2 | 10 | [https://doi.org/10.17602/M2/M699255](https://linkprotect.cudasvc.com/url?a=https%3a%2f%2fdoi.org%2f10.17602%2fM2%2fM699255&c=E,1,tJGAnW7QJL0NxWrlHvhOUo95wiT6sQ3B9-pRyiu-P20qGGphYuKFAXbUer99g3GXgHkS5UD-DYT9UBg0cACJJc4QsVqmFu2ICL5_aA63t5zJdRZpOMHllQ,,&typo=1) |
| Figs. 1d–f & 2a,b,e,f,j,k | X 1 | 0.16 | 340 | 2048  x 2048 | 84.75 | 16X | | 13.7 | 1 x 1 | n/a | [https://doi.org/10.17602/M2/M699258](https://linkprotect.cudasvc.com/url?a=https%3a%2f%2fdoi.org%2f10.17602%2fM2%2fM699258&c=E,1,Vjfn54SrueP4v4XWQ6gWqJFBPX5iXQ5GIbvcyU_TXePZ7aLQpB8WjlKwvu0fVsTncXWrVjoMTPoT5SYCGUjRnCAEgAVgZVprxflw8C6epJl8nNDNSw,,&typo=1) |
| Fig. 2c,d | X 4.44 | 0.03 | 96 | 2048  x 2048 | 23.75 | 16X | | 6.9 | 1 x 1 | n/a | [https://doi.org/10.17602/M2/M699261](https://linkprotect.cudasvc.com/url?a=https%3a%2f%2fdoi.org%2f10.17602%2fM2%2fM699261&c=E,1,-DU0BkwAvacIecBeENTsyFrtqMh0v7ZdQoQO7Jbf5a9_NMtaL2rKyGe3QYdh8mmwjcYFCwMFmzAQkX_L3R5Tw_3oM_XG3IQs-he09Nux9g,,&typo=1) |
